# A novel *Serratia* sp. ZS6 isolate derived from petroleum sludge secretes biosurfactant and lipase in medium with olive oil as sole carbon source

**DOI:** 10.1186/s13568-018-0698-9

**Published:** 2018-10-11

**Authors:** Xingcui Hu, Tao Cheng, Jianhua Liu

**Affiliations:** 1 Ocean College, Zhejiang University, Marine Science Building #379, 1 Zheda Road, Zhoushan, 316000 Zhejiang China; 2 Ocean Research Center of Zhoushan, Zhejiang University, Zhoushan, 316021 Zhejiang China

**Keywords:** 16S rDNA, Biosurfactant, In-gel lipase assay, Lipase, mT-RFLP, *Serratia* sp.

## Abstract

Biosurfactants emulsify lipids, facilitating their exposure to microorganisms in water. Secretion of biosurfactant together with lipase can enhance oil assimilation by microorganisms. In this study, we show the analysis of the biosurfactant and lipase producing microbe originating from oil-sludge. The enrichment of the biosurfactant-producing ZS6 strain from the mixed culture of sludge-derived microbes in medium with olive oil as sole carbon source was monitored using the modified T-RFLP (or mT-RFLP) methodology. Phylogenetic tree analysis based on 16S rDNA sequences suggested that ZS6 belonged to a member of *Serratia* sp. *Serratia* sp. ZS6 secreted both a serrawettin-type biosurfactant and lipase in medium with olive oil as sole carbon source. By using an in-gel lipase assay followed by LC–MS/MS analysis, we identified the amino acid sequences of the ZS6 lipase, which belonged to the lipase subfamily III of the family I. Its lipolytic activity was found to be enhanced by salinity, calcium, and methanol. Together, we show that the novel isolate *Serratia* sp. ZS6 secretes both biosurfactant and lipase which makes it useful for applications such as in food industry wastewater treatment and biodiesel production.

**Electronic supplementary material:**

The online version of this article (10.1186/s13568-018-0698-9) contains supplementary material, which is available to authorized users.

## Introduction

Biosurfactants are amphiphilic molecules such as fatty acids (Cooper et al. 1981; Knickerbocker et al. 2000), lipopeptides (Matsuyama et al. 1992; Seydlova and Svobodova 2008), glycolipids (Reiling et al. 1986; Van Bogaert et al. 2007), siderophore (Bodour et al. 2004; Rosenberg and Ron 1997), etc. that are present in plants and animals as well as microorganisms. Biosurfactants produced by microorganisms have attracted much attention for their diversity, performance under extreme conditions, possibility of large-scale production (Banat et al. 2000; Rahman et al. 2002).

Traditional methods of screening for biosurfactant-producing microorganisms are often involved in isolation of individual strains and subsequently testing for biosurfactant activity. Commonly used methods include, but not limited to, hemolytic assay (Mulligan et al. 1984; Rodrigues et al. 2006), bacterial adherence to hydrocarbons or BATH assay (Rosenberg et al. 1980), drop collapse test (Jain et al. 1991), oil spreading assay (Morikawa et al. 2000), emulsification assay (Rosenberg et al. 1979), and surface tension measurement. Given that biosurfactants may not be constitutively produced in microorganisms, these will either be missed during screening or multiply the work load for screening colonies under different growth conditions. We have previously modified the T-RFLP method (Liu et al. 1997) by using mini-PAGE gel to replace sequencing gel for monitoring dynamics of microbial populations without a DNA sequencer (Cheng et al. 2017). By using the modified T-RFLP (mT-RFLP) method to screen for biosurfactant-producing microorganisms capable of growing in MS medium supplemented with glucose as sole carbon source but not YE, we have isolated a *Pseudomonas* sp. ZS1 strain that produces up to 30 g L^−1^ rhamnolipids in medium with 2% glucose (Cheng et al. 2017).

Biosurfactants emulsify oils into micelles greatly expanding the interface between oil and water. Lipases (EC 3.1.1.3) are enzymes that hydrolyze fatty acyl ester bonds of acylglycerols at the interface between lipid and water (Lang 2002). Thus, secretion of both biosurfactant and lipase can enhance oil uptake by microorganisms (Colla et al. 2010; Ni’matuzahroh et al. 2015).

In this study, we show the isolation of a novel *Serratia* sp. ZS6 strain that produces both biosurfactant and lipase in medium containing oil. ZS6 assimilates 50% of total oil in just 32 h after growth at 30 °C in MS medium supplemented with 1% olive oil as sole carbon source. We propose that *Serratia* sp. ZS6 has potential for industrial usage.

## Materials and methods

### Strain isolation and culture manipulation

Petroleum sludge was collected from Sanjiang Ferry Terminal (30.141 N, 122.115 E), Zhoushan Archipelago, China in April 2016 and stored at 8 °C (Cheng et al. 2017). The purified ZS6 strain was deposited in the China General Microbiological Culture Collection Center with the accession number of CMGCC1.16530, whose 16S rDNA sequence was submitted to NCBI Genbank with the accession number of MF977715. Approx. 0.1 mg oil sludge was added to 3 mL MS medium (1 L contains: 0.6 g Na_2_HPO_4_, 0.2 g KH_2_PO_4_, 4.0 g NaNO_3_, 0.3 g MgSO_4_, 0.01 g CaCl_2_, 0.01 g FeSO) (Zajic and Supplisson 1972) supplemented with 2% yeast extract (Thermo Fisher Scientific Inc, Waltham, MA, USA) as nutrients for microbial growth at 30 °C with orbital shaking at 200 rpm min^−1^. The resulting culture was used as seed to inoculate in MS medium supplemented with 2% olive oil (Betis, Torres Y Ribelles SA, Dos Hermanas, Spain). Growth was determined by gravimetric method in which 15–50 mL culture was pelleted and washed with distill water twice using centrifugation at a relative centrifugal force of 12,000×*g* for 20 min (Eppendorf, Hamburg, Germany). Washed cell pellet was resuspended in 100 µL and transferred to pre-weighted glass fiber filter (GE Healthcare, Kent, UK) and dried in oven at 80 °C overnight. CDW was gravimetrically determined in triplicate using the AG204 balance (Mettler-Toledo Inc., Columbus, OH, USA).

### Analysis of biosurfactant activity in supernatant of cultures

Biosurfactant activity in supernatant was determined using oil spreading assay. Oil spreading assay follows the method previously described (Cheng et al. 2017).

### Preparation of biosurfactant crude extract from supernatant of ZS6 culture

Biosurfactant from ZS6 was extracted using the two-phase liquid–liquid separation method (Yuan et al. 2011). In brief, 1 mL supernatant of ZS6 culture was mixed with 0.2 mL ethanol followed by pH adjustment to 9 with addition of 1 M NaOH. Subsequently 0.5 g (NH_4_)_2_SO_4_ was added to the mixture and vortex to mix, then centrifuge at 4000×*g* for 5 min. The upper layer was transferred to a fresh tube and the lower layer was extracted with 0.2 mL ethanol again. The pooled upper layers were evaporated to result the crude extract. CMC of crude extract was determined using the BZY-B surface tensiometer (Fangrui Instrument Co. Ltd., Shanghai, China) with the du Nouy ring method (Butt et al. 2003). It was operated at 25 °C in triplicate or pentaplicate.

### PCR amplification of 16S rDNA sequences

Genomic DNA was extracted using Genomic DNA Extraction kit (Axygen Scientific Inc., Tewksbury, MA, USA) by following the manufacturer’s instruction. PCR amplification using 16S rDNA-specific primers 27F (5′-AGAGTTTGATCMTGGCTCAG-3′) and 1492R (5′-ACGGYTACCTTGTTACGACTT-3′) (Moreno et al. 2002) at the following condition: 5 min at 95 °C, then enter the cycle of denaturing at 95 °C for 30 s, annealing at 54 °C for 30 s, and extension at 72 °C for 90 s. After 30–40 cycles, the reaction was allowed to extend at 72 °C for additional 10 min. Single primer 27F also produces DNA fragments on template of ZS6 genomic DNA, which are not specific to 16S rDNA of ZS6 (see Additional file 1: Figure S1).

### Modified T-RFLP (mT-RFLP) analysis

To monitor the dynamic change of microbial populations in the mixed culture, we applied the mT-RFLP method (Cheng et al. 2017).

### Thin-layer chromatographic analysis

For analysis of biosurfactants in supernatant, crude extract derived from two-phase liquid–liquid separation method was dissolved in methanol and load on thin-layer chromatographic plate (Marine Biotech Co. Qingdao, China). It was developed in chloroform, methanol, 5 M ammonia (80:25:4, v/v/v). The plate was exposed to iodine vapor for 2 min. Acid hydrolysis was performed in a high temperature resistant glass vessel containing 1 mL 6 N HCl at 110 °C for 1 h. Ninhydrin staining with or without acid hydrolysis was performed using 0.5% ninhydrin in acetone (Aladdin reagents Co. Ltd, Shanghai, China).

For analysis of lipids in supernatant, 40 mL supernatant was extracted with hexane. After evaporation, lipids were weighted and dissolved in hexane and loaded on TLC plate (Marine Biotech Co.). Plate was developed in two phases: first chloroform and methanol (5:1, v/v) was used to develop to one-third of the plate length. After drying, the plate was continued to develop in mixture of hexane, diethyl ester, and acetic acid (7:3:01, v/v/v) to the remaining two-third of the plate. The scanned image of TLC plate was subjected to quantification of TAG, FFA, and DAG and MAG using ImageJ (imagej.nih.gov).

### Lipase activity assay

Substrate pNPB was used in lipase activity assay. It was based on a previously reported procedure (Ghati and Paul 2015) with minor modification. In brief, 0.625 mL cell free supernatant of ZS6 culture was mixed with equal volume of 0.2 M phosphate buffer (pH 7.2), pre-warm at 30 °C for 10 min. Subsequently 50 µL of 100 mM pNPB was added and incubated at 30 °C for 10 min. The reaction was kept on ice for 10 min, and centrifuge at 2000×*g* at 4 °C for 10 min. Supernatant was taken and subjected to measurement of OD at the wave length of 405 nm. The concentration of the lipase in U (one lipase unit was defined as liberating 1 µmol of butyric acid min^−1^ at 30 °C, pH 7) was obtained based on the standard curve using pNP.

To test lipase activities at various temperatures, reactions was carried out at the designated temperature; for activities at various pH values, pH of the reaction solution was adjusted using sodium acetate buffer at the range between pH 4 and pH 5, potassium phosphate buffer at the range between pH 6 and pH 8, glycine–NaOH buffer at the range between pH 9 and pH 10; for various salinities, NaCl was added to the reaction solution at the various designated concentrations; for metal ions, corresponding chemicals were added to the reaction solution at the final concentration of 2 mM; for various organic solvents, individual organic solvents were added to the reaction solution at the final concentration of 5% (v/v). All measurements were performed in triplicate.

### Preparation of total proteins from supernatant

Approx. 250 mL cell-free supernatant of the ZS6 culture was lyophilized using the Freezer Dryer Lyophilizer (Labconco Cor. Kansas City, MO, USA) for 1 day. Lyophilized powder was dissolved in 20 mM Tris–HCl and dialyzed against 20 mM Tris–HCl overnight using dialysis tubing with the size of the pores ranging from 8 to 14 kD (Sinopharm Chemical Reagent Co. Ltd, Shanghai, China) at 4 °C.

### Native PAGE gel electrophoresis and in-gel lipase assay

Protein analysis using native PAGE gel electrophoresis was followed by the protocol in Molecular Biology, a laboratory manual (Green and Sambrook 2012). Native PAGE gel containing lipase proteins was washed in 50 mM Tris–HCl (pH 8) and overlaid onto a 2% agarose gel containing 0.01% phenol red (Aladdin Industrial Co., Shanghai, China), 1% olive oil, and 10 mM CaCl at pH 7.4 for 30 min at 40 °C. Position containing lipase activity was indicated by yellow spot from the pink background (Singh et al. 2006).

### Protein sequencing analysis

Protein in PAGE gel at the position was excised and trypsinized using sequencing grade modified trypsin (Promega Co. Madison, WI, USA). The resulting trypsinized peptides were subjected to nanoLC-ESI–MS/MS analysis (Thermo Fisher Scientific Inc.) in ProTech (Protech Co. Suzhou, China).

### Lipase gene sequence analysis

Based on primers from E13 *lipA* gene sequences (KJ868240.1) encoding the lipase protein that matched with 12 trypsinized peptides failed to amplify any DNA fragment using PCR on template of ZS6 genomic DNA. To find the potential variations in DNA sequences, three sequences shared homologous to *lipA* (CP011303.1, CP014017.2, EF202840.1) were aligned using ClustalW (http://www.clustal.org). PCR primers (ZS6-lipA-F, 5′-ATGGGAATCTTTAATTATCAAGG-3′; ZS6-lipA-R, 5′-TTAGGCCAGTACCACYTGGCCG-3′) derived from aligned sequences that differed from E13 *lipA* produced a 1.8 kb fragment (deposited in NCBI Genbank with an accession number of MG897498.1) (see Additional file 1: Figure S2).

### Quantitative reverse transcription PCR analysis

To investigate changes of *lipA* transcription levels during ZS6 growth in MS medium supplemented with 2% olive oil, quantitative reverse transcription PCR (qRT-PCR) assay using primers ZS6-lipA-RTF (5′-GCACCACAAGAGTCCGCTAC-3′) and ZS6-lipA-RTR (5′-TTCAATACCCGCATCAATCC-3′) was performed, in which transcription of rplU with primers (rplU-F, 5′-GTGGTAAACAACACCGAGTAAG-3′; rplU-R, 5′-CAACGAAAGGAACGCCGATT-3′) in ZS6 was used as reference (Petersen and Tisa 2014). qRT-PCR was performed as follows: total RNA of 500 ng was used for the first strand cDNA synthesis with PrimeScript RT reagent kit plus gDNA Eraser (Takara Bio Inc., Tokyo, Japan) according to manufacturer’s protocol. The resulting cDNA was tenfold diluted and subjected to quantitative PCR using SYBR Premix Ex Taq II kit (Takara Bio Inc.) on a Roche LightCycler 480 instrument (Roche, Basel, Switzerland) with the condition as follows: initial denaturing for 30 s at 95 °C, followed by 40 cycles of 5 s at 95 °C and 30 s at 58 °C.

## Results

### Isolation of biosurfactant-producing microbes in medium with olive oil as sole carbon source

**Fig. 1:**
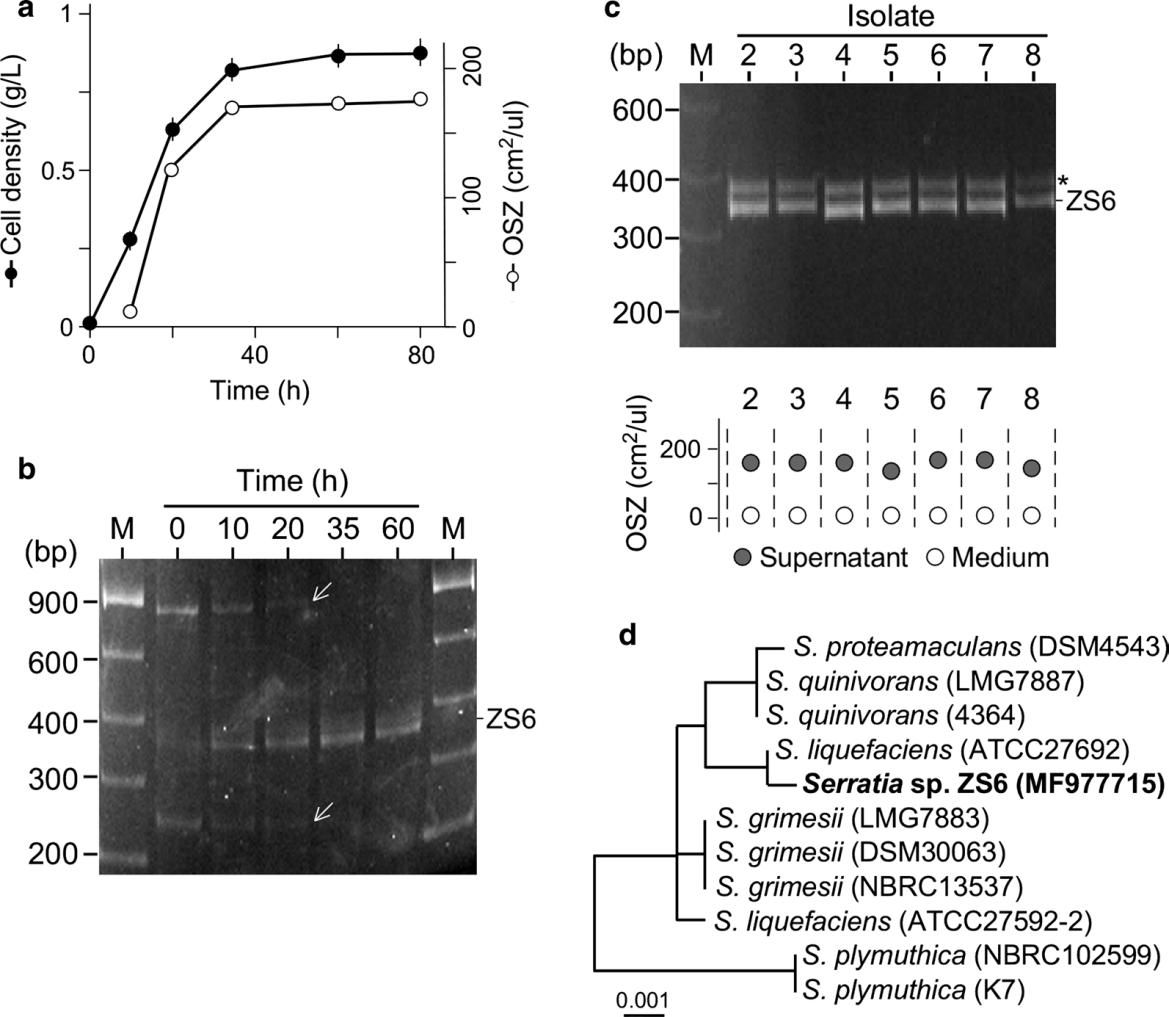
Enrichment and isolation of the oil emulsifying microbe in medium with olive oil as sole carbon source. **a** Cell growth is associated with production of biosurfactant activity. X- and Y-axes indicate time (h) and cell density (g L^−1^, solid circle) or oil spreading zone (OSZ, cm^2^ µL^−1^, open circle). **b** Microbial populations enriched in cultures with olive oil as sole carbon source. mT-RFLP analysis shows the level changes of three major populations (or fragments), two of which decrease (see arrows) and one increase (see ZS6). **c** mT-RFLP pattern of individual isolates. A non-specific fragment (see asterisk) is found to be associated with 16 rDNA PCR fragment in ZS6. Biosurfactant activity of individual isolates is shown in the bottom panel. **d** The phylogenetic tree analysis is based on 16S rDNA sequences whose GenBank accession number (ZS6, MF977715) is shown in parentheses. ZS6 isolate belongs to the member of *Serratia* spp.

We wanted to investigate whether microorganisms derived from petroleum sludge would grow in MS medium supplemented with 2% olive oil as sole carbon source. For this reason, petroleum sludge-derived microbes were cultivated in MS medium supplemented with olive oil. Using a gravimetric method, we found that the maximum cell density was ~ 0.75 g L^−1^ based on triplicate (Fig. 1a, solid circle). Meanwhile, biosurfactant concentration measured by oil-spreading zone using cell-free supernatant was found to be ~ 175 cm^2^ μL^−1^ at its maximal level (Fig. 1a, open circle). Hence, we concluded that biosurfactant-producing microbes were enriched in culture 60 h after growth in MS medium supplemented with olive oil.

To investigate the enriched microbial populations in culture, we performed the analysis using a modified T-RFLP (or mT-RFLP) method (Cheng et al. 2017). In mT-RFLP analysis, each fragment would represent a microbial population. We found that two mT-RFLP fragments of ~ 850 bps and 240 bps in length were predominated in initial culture (i.e., at 0 h), but failed to propagate 20 h after growth (Fig. 1b, see arrow). On the other hand, a fragment of 370 bps in length appeared and dominated at 60 h after growth, suggesting a potential strain responsible for producing biosurfactant activity.

To isolate strains producing biosurfactant, colonies derived from culture 60 h after growth were randomly selected and subjected to mT-RFLP analysis. We found that all isolates contained a mT-RFLP fragment of 370 bps in length and exhibited biosurfactant production (Fig. 1c). However, we noted that an additional fragment of 400 bps in length co-occurred in all isolates. PCR analysis using single primer indicated that forward 27F but not reverse 1492R primer was able to produce fragments, suggesting a non-specific amplification of 16S rDNA using ZS6 genomic DNA as template (see Additional file 1: Figure S1). Sequencing analysis of the 16S rDNA bearing a HhaI site at 370 bps from the 5′ end indicated that ZS6 belonged to a member of *Serratia* sp. (Fig. 1d).

### ZS6 produces a serrawettin-type biosurfactant in MS medium supplemented with olive oil but not yeast extract

**Fig. 2:**
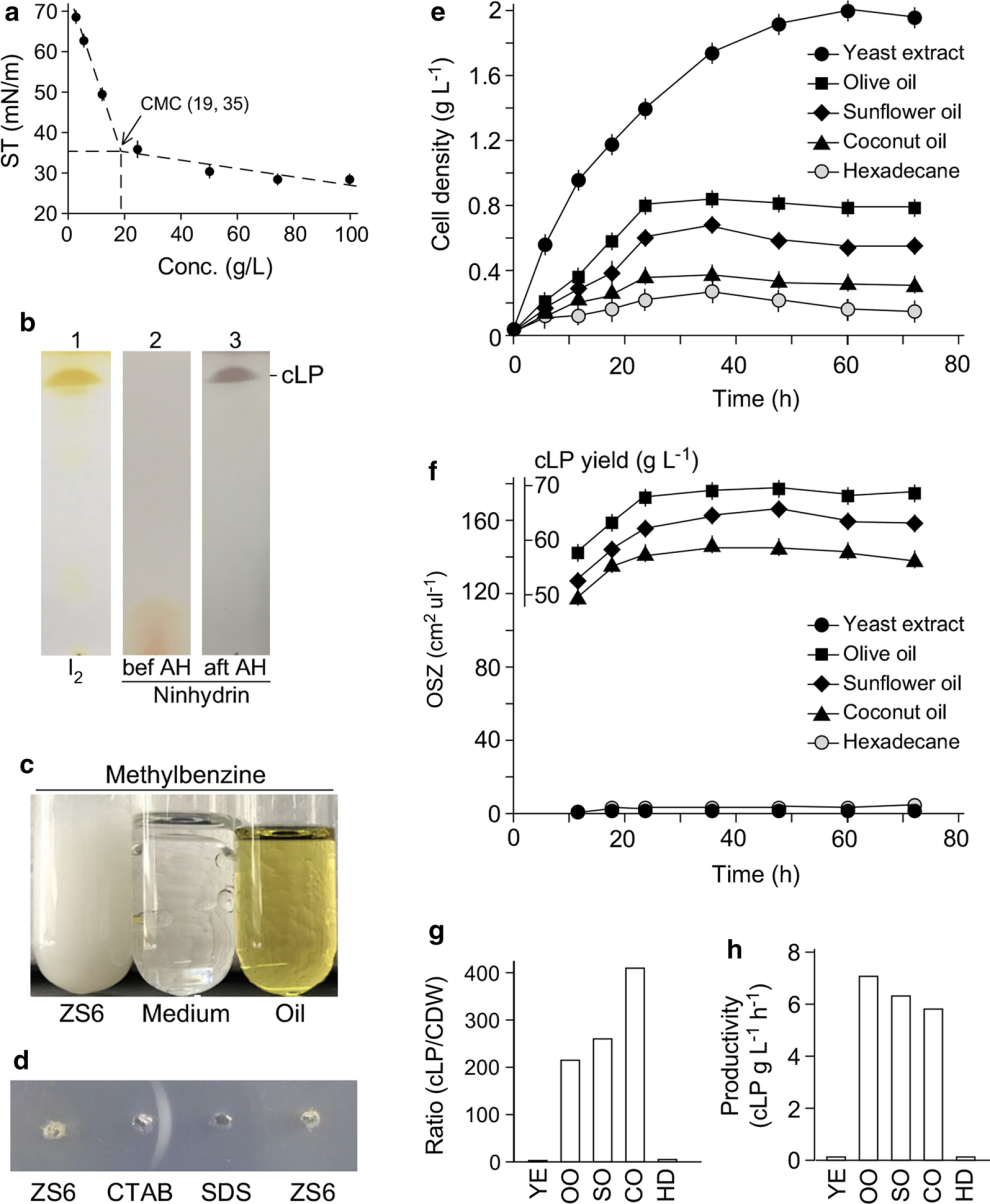
A serrawettin-type biosurfactant from ZS6 is found at high-level when cultivated in olive oil medium. **a** CMC analysis of crude extract derived from supernatant of ZS6 culture. X- and Y-axes indicate concentrations of biosurfactant crude extract (g L^−1^) and surface tension ST (mN m^−1^). **b** Thin-layer chromatographic analysis of the biosurfactant crude extract. Lanes 1, 2, and 3 show extract of iodine vapor staining, ninhydrin staining prior to (bef) and after (aft) acid hydrolysis (AH), respectively. **c** High capability of methylbenzene emulsification. Images show 24 h after mixing the supernatant of ZS6 culture (super), blank medium, and olive oil with methylbenzene (6:1 v/v). **d** Image shows the result of agar gel immunodiffusion assay. ZS6 biosurfactant is a nonionic cyclolipopeptide. SDS and CTAB are anionic and cationic surfactants, respectively. **e** Growth curve analysis of ZS6 under various growth conditions indicated. **f** Biosurfactant production from ZS6 under various growth conditions. **g** Efficiency of biosurfactant production per unit of cell mass under various growth conditions. The ratio between level of cyclic lipopeptide (cLP) or OSZ and CDW is based on measurements at 36 h after growth. YE for yeast extract, OO for olive oil, SO for sunflower oil, CO for coconut oil, and HD for hexadecane. **h** Productivity of biosurfactant in ZS6 under various growth conditions. The display is identical to **g**

We wanted to investigate the type of biosurfactant produced from the *Serratia* sp. ZS6 strain. For this reason, cell-free supernatant of ZS6 culture was subjected to the two-phase extraction methodology that was adapted for lipopeptides (Yuan et al. 2011). Crude extract exhibited the critical micelle concentration (CMC) of 19 g L^−1^ that reduced surface tension to 35 mN m^−1^ (Fig. 2a). Thin-layer chromatographic (TLC) analysis showed a positive staining with iodine vapor, suggesting a probable lipid moiety (Fig. 2b, lane 1). On the other hand, ninhydrin (2,2-dihydroxyindane-1,3-dione) staining was not apparent prior to but after acid hydrolysis, suggesting a potential cyclopeptide moiety (Fig. 2b, lanes 2 and 3). The emulsification index E24 of the ZS6 biosurfactant against methylbenzene was 100% (Fig. 2c). Agar gel immunodiffusion assay indicated that biosurfactant from ZS6 was nonionic (Fig. 2d). These results suggested that the ZS6 biosurfactant was a serrawettin-type of cyclolipopeptides.

Subsequently, we wanted to investigate the productivity of biosurfactant under various growth conditions. For this reason, the ZS6 strain was subjected to growth in MS medium supplemented with olive oil, sunflower oil, coconut oil, hexadecane, and yeast extract. We found that yeast extract gave rise to the highest maximum cell density of 2 g L^−1^ (Fig. 2e, black circle). On the other hand, the hexadecane produced the lowest cell mass of 0.2 g L^−1^ (Fig. 2e, grey circle). Olive oil, sunflower oil, and coconut oil produced cell biomasses of 0.8 g L^−1^, 0.6 g L^−1^, and 0.3 g L^−1^, respectively.

Though the highest biomass was found when cultured in YE medium, the biosurfactant activity was hardly detected, whose level was similar to that in hexadecane (Fig. 2f, black and grey circles). On the other hand, high level of biosurfactant production was detected when cultured in olive oil, sunflower oil, and coconut oil media: activities were equivalent to supernatant crude extract of 70 g L^−1^, 65 g L^−1^, and 57 g L^−1^, respectively (Fig. 2f). The ratio between biosurfactant mass and cell biomass would indicate the efficiency of biosurfactant production per unit of cell mass under the growth condition. To this end, at the onset of stationary phase or 24 h after growth, we found that coconut oil exhibited the highest ratio of 400 between biosurfactant mass and cell biomass, which was twofold and 1.5-fold higher than that of sun flower oil and olive oil, respectively (Fig. 2g). On the other hand, ZS6 in olive oil medium exhibited the highest productivity of biosurfactant at a level of 7 g L^−1^ h^−1^ (Fig. 2h).

### Assimilation rate of olive oil by ZS6

**Fig. 3:**
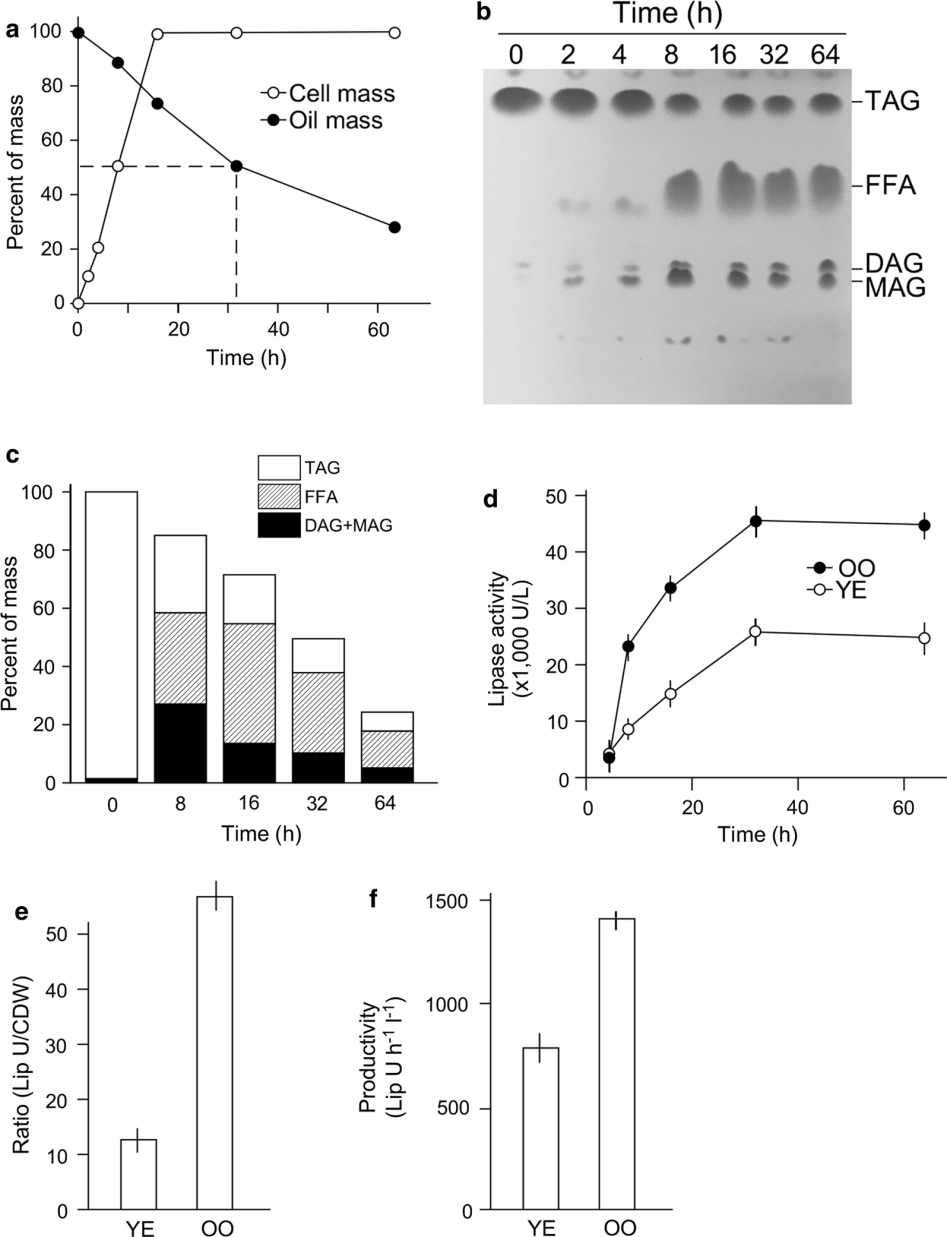
Characterization of olive oil consumption by ZS6. **a** Gravimetric analysis of oil assimilation by ZS6. X- and Y-axes indicate the time (h) and percent of cell mass (closed circle) and oil mass (open circle). **b** Thin-layer chromatographic analysis of oil residues extracted from supernatant of ZS6 cultures at various time points indicated. Equal amount of hexane extracts is loaded on TLC plate. Separated fractions of compounds are deduced based on their molecular weight determined using MS analysis. *TAG* triacylglycerol, *FFA* free fatty acid, *DAG* diacylglycerol, *MAG* monoacylglycerol. **c** Changes of lipid compositions in culture. Total lipids are mainly consistent of TAG (open rectangle), FFA (stripped rectangle), DAG and MAG (black rectangle). **d** ZS6 lipase activity in MS medium supplemented with olive oil and YE. X- and Y- axes indicate time (h) and lipase activity (U L^−1^). Closed and open circles indicate cells in medium supplemented with olive oil (OO) and YE, respectively. **e** Efficiency of lipase production per unit of cell mass. Ratio of maximum lipase activity in U and CDW. **f** Productivity of lipase activity in U per hour per liter

We investigated the assimilation rate of olive oil by ZS6 in MS medium supplemented with 1% olive oil using gravimetric methodology. We found that 50% of the olive oil (or 5 g in 1 L of medium) was assimilated in 32 h after growth (Fig. 3a). Subsequently, we investigated the rate of triacylglycerol (TAG) hydrolysis in culture. To this end, oil extracted from supernatant using hexane was subjected to TLC analysis (Fig. 3b). Equal amount of extracted oils from supernatant of cultures at various time points after growth was loaded on TLC plate and developed using organic solvents. The TLC plate was scanned and quantified using Image J (imageJ.nih.gov). We found that, of a total of 10 g TAG prior to grow in 1 L of medium, ~ 1.5 g of TAG was consumed 8 h after growth (Fig. 3c, open rectangle). Of the remaining 8.5 g of oil, TAG was only account for 2.7 g (31.75%). The weight of 3.1 g (36.5%) and 2.7 g (31.75%) was free fatty acid (FFA) and diacylglycerol (DAG) and monoacylglycerol (MAG), respectively (Fig. 3c, striped and black rectangles). These results suggested that the free fatty acids liberated from TAG by lipase were not immediately assimilated by ZS6 during growth.

To investigate lipase activity in supernatant of cultures, we performed colorimetric assay for lipase activity using pNPB as substrate (Ghati and Paul 2015). We found that the maximum lipase activity of 45,000 U L^−1^ in supernatant occurred at 32 h after growth in MS medium supplemented with 2% olive oil (Fig. 3c, close circle). On the other hand, maximum lipase activity of 25,000 U L^−1^ in supernatant at 32 h after growth was found in MS medium supplemented with YE (Fig. 3c, open circle), though no TAG was detected in YE medium. Efficiency of lipase production per unit of cell dry mass in medium supplemented with olive oil and YE was 56.2 U CDW^−1^ and 12.5 U CDW^−1^, respectively (Fig. 3e). Productivity of lipase activity in medium supplemented with olive oil and YE was 1400 U h^−1^ L^−1^ and 780 U h^−1^ L^−1^, respectively (Fig. 3f).

### Identification of ZS6 lipase protein sequences

**Fig. 4:**
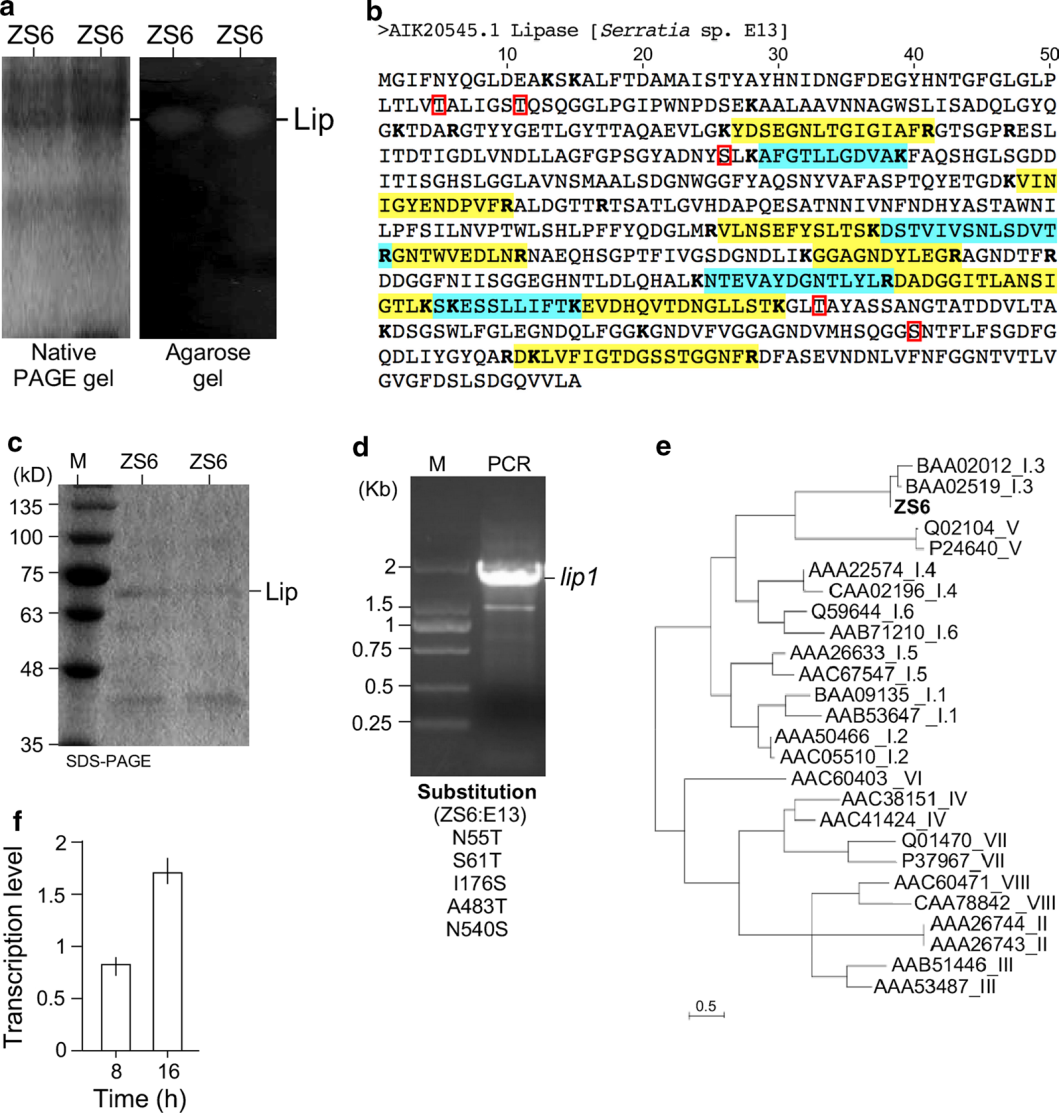
Identification of lipase protein and gene sequences in ZS6. **a** In-gel lipase assay. Left panel shows the native PAGE analysis of proteins prepared from supernatant of ZS6 culture. The right panel shows the in-gel analysis of lipases. Bright spots indicate the increase of acidity caused by lipid hydrolysis. **b** Lipase shared with trypsinized peptide sequences generated in LC–MS/MS analysis. Residue at the end of trypsin peptides were highlighted in bold. Peptide sequences identified by MS analysis are highlighted. Residue at the end of trypsin peptides were highlighted in bold. **c** Probable lipase protein in SDS-PAGE gel. **d** Probable lipase encoding DNA is amplified using PCR analysis. Amino acid substitutes between E13 Lip and ZS6 Lip are shown in the bottom panel. Five amino acid substitutions were found in the deduced protein sequences compared with that of the E13 LipA. **e** Sequence-based lipase phylogenetic tree analysis. Sequences whose Genbank accession number (ZS6, MG897498.1) followed by the lipase family [I–VIII families (Arpigny and Jaeger 1999)] is shown. **g** Levels of lipase gene expression at 8 h and 16 h after growth based on RT-qPCR analysis are shown

To identify the putative lipase secreted from ZS6, the lyophilized supernatant was resuspended and subjected to native PAGE gel electrophoresis (Fig. 4a, left panel). The gel was subsequently overlaid to an agarose gel containing olive oil and phenol red as pH indicator. Hydrolysis of oil led to the decrease of pH, which changed the color of the pH indicator. We found that one of the bands in PAGE gel showed to produce yellow spot on overlaid agarose gel with the pink background (Fig. 4a, right panel). Proteins in gel at that position was cut out and subjected to trypsin digestion and LC–MS/MS analysis. Twelve distinct peptides revealed from LC–MS/MS analysis were found to match perfectly to the LipA from *Serratia* sp. E13 (Fig. 4b) (Šiekštele et al. 2015). Analysis of the ZS6 supernatant proteins revealed a probable lipase with a molecular weight between 63 and 75 kD in SDS-PAGE (Fig. 4c).

To investigate the gene encoding for the ZS6 LipA, we first tried to amplify the DNA sequences using primers derived from the E13 *lipA* gene (KJ868240.1). But it failed to produce any DNA fragment. To find possible variations at the 3′ end and 5′ end of the *lipA* gene sequences, three *lipA* homologous sequences were obtained using the basic local alignment search tool (BLAST) (http://blast.ncbi.nlm.nih.gov). Alignment analysis of homologous sequences indicated that there were two types of sequences at the 5′ end and 3′ end of the *lipA*-like genes (see Additional file 1: Figure S2). After using another type of DNA sequences as primers, we were able to amplify a fragment of ~ 1.8 kb in size (MG897498.1) (Fig. 4d). Deduced amino acid sequences from the DNA appeared to share 99.1% of identity to that of E13 LipA with five amino-acid substitutions. Sequence-based phylogenetic analysis indicated that ZS6 LipA belonged to the subfamily III of the family I, the true lipase family (Fig. 4e) (Arpigny and Jaeger 1999).

It was shown that lipase activity increased upon cell growth (see Fig. 3d). Consistent with that, we found that the transcription level of lipase encoding gene ZS6 *lipA* was increased from 0.8 arbitrary unit (a.u.) at 8 h to 1.7 a.u. at 16 h (Fig. 4g). These results suggested that lipase activity was transcriptionally regulated in *Serratia* sp. ZS6.

### Lipase activity from ZS6 is enhanced by salinity, calcium, and methanol

**Fig. 5:**
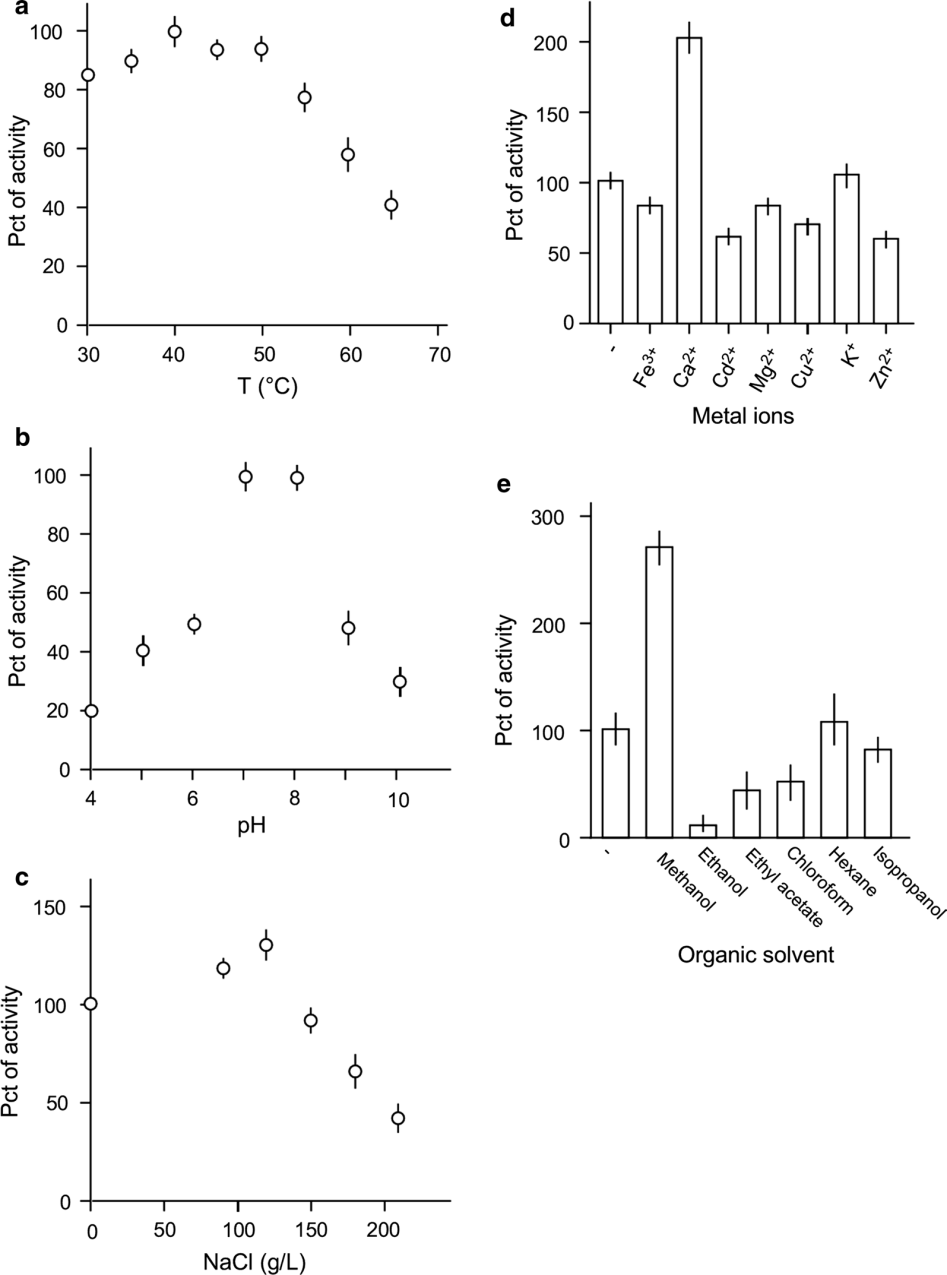
ZS6 lipase activity at various conditions. Initial activity is based on the condition at 37 °C, pH 7, without salinity, and no additional metal ions or organic solvent. Lipase activity under conditions of various temperatures (**a**), acidities (**b**), salinities (**c**), metal ion additions (**d**), and organic solvents (**e**)

To investigate factors that affected the activity of lipase from ZS6, lipase in supernatant was subjected to colorimetric assay for activity under various conditions. To this end, lipase activity under the reaction conditions at 37 °C, pH 7, without NaCl, without metal ions such as Fe^3+^, Ca^2+^, Cd^2+^, Mg^2+^, Cu^2+^, K^+^, and Zn^2+^, and without organic solvent was set as reference for comparison. At this point, we found that the ZS6 lipase activity was above 80% within the temperature range from 30 °C to 50 °C (Fig. 5a). ZS6 lipase displayed the optimal activity at pH 7 and pH 8 (Fig. 5b). Notably, ZS6 lipase activity increased by 40% at 120 g L^−1^ NaCl (Fig. 5c), suitable for utility under high salt conditions. Subsequently, we tested seven metal ions such as Fe^3+^, Ca^2+^, Cd^2+^, Mg^2+^, Cu^2+^, K^+^, and Zn^2+^ at the concentration of 2 mM and found that lipase activity was increased by twofold in presence of Ca^2+^ (Fig. 5d). ZS6 lipase activity was increased by 2.7-fold in 5% methanol and decreased by twofold or greater in 5% chloroform, ethyl acetate, and ethanol (Fig. 5e). On the other hand, the lipase activity was hardly altered in 5% hexane or isopropanol.

## Discussion

In this study, we show the isolation of the biosurfactant-producing *Serratia* sp. ZS6 strain after enrichment through growth in MS medium supplemented with olive oil (see Fig. 1). By using the mT-RFLP method, enrichment of microorganisms is readily detected. Meanwhile, we show that the common primer 27F for PCR amplification of 16S rDNA (Moreno et al. 2002) produces the non-specific fragment on template DNA derived from ZS6 cells.

*Serratia* spp. are known to produce serrawettin, a nonionic lipopeptide type biosurfactants (Matsuyama et al. 1985). In this analysis, we show that the ZS6-producing biosurfactant on TLC plate is stained using ninhydrin method only after but not prior to acid hydrolysis, suggesting a cyclolipopeptide nature (see Fig. 2). Furthermore, the biosurfactant exhibits a characteristic of nonionic molecule, resembling serrawettin.

The productivity of biosurfactant crude extract produced from ZS6 is 7 g L^−1^ h^−1^, the highest in MS medium supplemented with olive oil (see Fig. 2). Nevertheless, we failed to confirm the structure of serrawettin-like biosurfactant from the crude extract using LC–MS/MS analysis. Hence, we propose that ZS6 strain produces the putative serrawettin-type biosurfactant.

We find that ZS6 consumes 50% olive oil in just 32 h after growth, indicating that oil assimilation rate is rapid in ZS6 cells (see Fig. 3). Lipase assay indicates that ZS6 secretes lipase to its 50% maximum activity 8 h after growth, suggesting that the high efficiency of oil internalization is partly attributed to the extracellular lipase. Simultaneous secretion of biosurfactant and lipase is known to enhance oil assimilation by microorganisms (Colla et al. 2010; Ni’matuzahroh et al. 2015). This result implies that ZS6 is a useful strain for treatment of food industry wastewater that often contains high concentrations of lipids.

We show that ZS6 hardly secretes biosurfactant in MS medium supplemented with YE (see Fig. 2). On the other hand, lipase is secreted in YE medium in which no TAG is detected (see Fig. 3). The maximum level in YE medium is 55% of that in olive oil medium, suggesting that production of surfactant but not lipase requires the induction of vegetable oils in ZS6.

By using in-gel lipase assay followed by LC–MS/MS analysis, we show, in this study, that ZS6 lipase belongs to the subfamily III of the family I, the true lipase family (see Fig. 4) (Arpigny and Jaeger 1999). Activity of lipase in subfamily III does not need helper proteins for function as those in subfamily I and II of the true lipase (Arpigny and Jaeger 1999), applicable for various industrial applications. In addition, the ZS6 LipA exhibits methanol enhancement, making it favorable for production of biodiesel, methyl esters of fatty acids that are often produced by base catalyzed transesterification of triacylglycerol with methanol (Jaeger and Eggert 2002).

In conclusion, we show, in this study, that isolation of a novel oil-eating microorganism ZS6 is assisted through the enrichment in oil containing medium. The enrichment monitored by the mT-RFLP analysis facilitates the isolation. ZS6 belongs to a member of *Serratia* sp. based on the 16S rDNA sequence-based analysis. It secretes both serrawettin-type biosurfactant and lipase and is able to assimilate 50% olive oil in medium just 32 h after growth. Using the in-gel assay followed by LC–MS/MS analysis, lipase protein sequence identified resembles those in the subfamily III of the family I, whose activity requires no help factors. Additionally, we show that ZS6 lipase is enhanced by salinity, calcium, and methanol. Hence, we propose that ZS6 is a useful strain for industrial applications such as food industry wastewater treatment and biodiesel production.

## Additional file

**Additional file 1: Figure S1.** Non-16S rDNA amplification by 27-F primer. Pri stands for primer; Iso for isolate; asterisk for primer dimers. (A) Single primer PCR assay. PCR reaction containing single primer 27-F or 1492-R using the condition identical to 16S rDNA amplification with a pair of primers 27-F and 1492-R. (B) mT-RFLP analysis using fluorescence labeled 1492-R primer. **Figure S2.** DNA primers deduced from lipase A genes 5′-end of the lipase DNA sequence.

## Abbreviations

CDW: cell dry weight
CMC: critical micelle concentration
LC–MS/MS: liquid chromatography coupled with tandem mass spectrometry
MS: mineral salt
mT-RFLP: modified terminal-labeled restriction fragment length polymorphism
OSZ: oil-spreading zone
pNPB: *p*-nitrophenyl butyrate
TLC: thin-layer chromatography
