# Supplementary material for: A novel Serratia sp. ZS6 isolate derived from petroleum sludge secretes biosurfactant and lipase in medium with olive oil as sole carbon source
Source: AMB Express. 2018 Oct 11;8:165. doi: 10.1186/s13568-018-0698-9 (PMC6182024; doi:10.1186/s13568-018-0698-9)
Supplement: Supplementary file 1 — Additional file 1: Figure S1. Non-16S rDNA amplification by 27-F primer. Pri stands for primer; Iso for isolate; asterisk for primer dimers. (A) Single primer PCR assay. PCR reaction containing single primer 27-F or 1492-R using the condition identical to 16S rDNA amplification with a pair of primers 27-F and 1492-R. (B) mT-RFLP analysis using fluorescence labeled 1492-R primer. Figure S2. DNA primers deduced from lipase A genes 5′-end of the lipase DNA sequence. [file 13568_2018_698_MOESM1_ESM.pdf]

Additional File:

A novel *Serratia* sp. ZS6 isolate derived from petroleum sludge secretes biosurfactant and lipase in medium with olive oil as sole carbon source

Xingcui Hu[1], Tao Cheng[1], and Jianhua Liu[1,2*]

[1]Ocean College, Zhejiang University, Zhoushan, ZJ 316000, China

[2]Ocean Research Center of Zhoushan, Zhejiang University, Zhoushan, ZJ 316021, China

[*]Corresponding author

Additional file 1: Figure S1. Non-16S rDNA amplification by 27-F primer. Pri stands for primer; Iso for isolate; asterisk for primer dimers. (A) Single primer PCR assay. PCR reaction containing single primer 27-F or 1492-R using the condition identical to 16S rDNA amplification with a pair of primers 27-F and 1492-R. (B) mT-RFLP analysis using fluorescence labeled 1492-R primer.

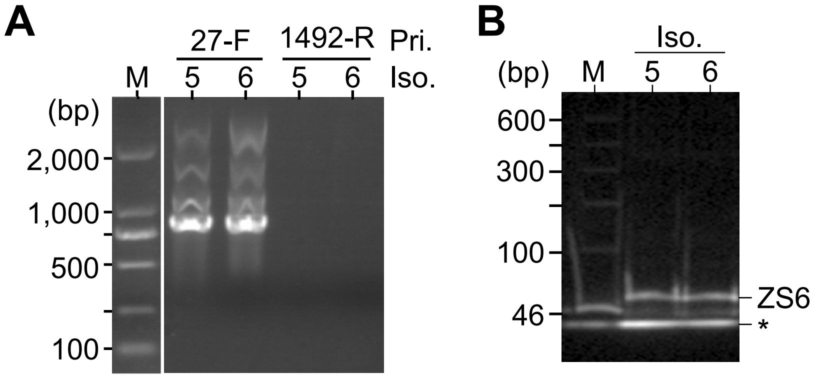

Additional file 1: Figure S2. DNA primers deduced from lipase A genes

5'-end of the lipase DNA sequence:

```
              *********** ***** ***** ************** ****** *************
1. CP011303.1 ATGGGAATCTTTAATTATCAAGGCCTCGATGAGGCGAAGTCCAAAGCGTTATTTACCGAT
2. CP014017.2 ATGGGAATCTTTAATTATCAAGGCCTCGATGAGGCGAAGTCCAAAGCGTTATTTACCGAT
3. EF202840.1 ATGGGAATCTTTAATTATCAAGGTCTCGATGAGGCGAAATCCAAAACGTTATTTACCGAC
4. KJ868240.1 ATGGGAATCTTCAATTACCAAGGGCTCGATGAGGCGAAGTCCAAAGCGTTATTTACCGAT
              1.......10........20........30........40........50........60
              ATGGGAATCTTTAATTATCAAGG
```

3'-end of the lipase DNA sequences:

```
              *** **** *********************** ***** *****
1. CP011303.1 GGGGTCGGGTTCGATAGCCTGTCGGACGGCCAGGTGGTACTGGC---- 1848
2. CP014017.2 GGGGTCGGGTTCGATAGCCTGTCGGACGGCCAGGTGGTACTGGC---- 1848
3. EF202840.1 GGGATCGGATTCGATAGCCTGTCGGACGGCCAAGTGGTACTGGCCTAA 1848
4. KJ868240.1 GGGGTCGGGTTCGATAGCCTGTCGGACGGCCAGGTGGTGCTGGCGTAA 1848
              ......1810......1820......1830......1840........
                                          CGGCCARGTGGTACTGGCCTAA
```

Note: Identical nucleotides in four different lipase sequences are indicated by asterisk (*). Primer sequences based on 4. *KJ868240.1* rDNA (see sequences in red) were unable to amplify lipase gene fragment using ZS6 genomic DNA as template. This could be caused by different sequences at the termini of lipase genes in different strains. Hence, primer sequences based on the upper three lipase genes (see underlined) were used in PCR amplification of lipase gene in ZS6, which was successful.

Primers that successfully produced LipA coding sequences in ZS6 are showing below:

Fwd, 5'-ATGGGAATCTTTAATTATCAAGG-3'

Rev, 5'-TTAGGCCAGTACCACYTGGCCG-3'
